# Factors Influencing Unintended Pregnancies amongst Adolescent Girls and Young Women in Cambodia

**DOI:** 10.3390/ijerph16204006

**Published:** 2019-10-19

**Authors:** Farwa Rizvi, Joanne Williams, Elizabeth Hoban

**Affiliations:** School of Health and Social Development, Deakin University, 221 Burwood Highway, Burwood, VIC 3125, Australia; jo.williams@deakin.edu.au (J.W.); elizabeth.hoban@deakin.edu.au (E.H.)

**Keywords:** adolescents, reproductive and sexual health, unintended pregnancies, contraception, women’s health, women’s status

## Abstract

*Background:* Unintended pregnancies in Cambodian youth are a major reproductive health concern with detrimental personal and socioeconomic consequences. A social ecological model was used to identify sociodemographic factors potentially associated with unintended pregnancies, and an analysis of data from the 2014 Cambodian Demographic and Health Survey was used to determine associations. *Methods:* Weighted data were analysed using multiple logistic regression analyses for 3406 Cambodian sexually active single, in union or married females aged 15–29 years. *Results:* The prevalence of unintended pregnancy was 12.3%. Unintended pregnancy was significantly associated with younger age groups (15–24 years), multiparity, history of abortion, and current use of modern contraceptive methods. All women had an increased likelihood of unintended pregnancy when the husband alone or someone else in the household made decisions about their access to healthcare. *Conclusion:* The burden of unintended pregnancies is associated with young age, multiparity, history of abortions, unemployment, and low autonomy for accessing healthcare. Multi-pronged, holistic reproductive and sexual health program interventions are needed to increase literacy and accessibility to modern contraception and to raise awareness about women’s health and status in Cambodia.

## 1. Introduction

Unintended pregnancy (UIP) and abortion amongst Cambodian adolescent girls and women in their twenties are fast becoming major public health issues due to the negative personal, social, and economic implications for both mother and child [1]. Cambodia is home to the largest adolescent and young adult population in the Southeast Asian region, with two thirds of the 14.7 million population being under the age of 30 years [1,2], thus providing the potential for economic and social development for demographic dividends [2].

Cambodian youth face numerous obstacles to sound sexual and reproductive health (SRH) including lack of SRH literacy and limited access to modern contraceptive methods [3]. Cambodia is lagging far behind neighbouring countries in the implementation of effective strategies for improving sexual and reproductive health (SRH) [4,5]. This may be partly explained by Cambodia’s political history. During the Khmer Rouge Regime’s genocide of more than two million people in the 70s, the country’s health infrastructure was decimated and there was very slow recovery over the next two decades [4].

Cambodia adopted the Birth Spacing policy in 1995 as part of the family planning (FP) program to reduce unintended pregnancies and maternal and neonatal mortality [6]. Cambodian national policy and strategies on safe motherhood were introduced in 1997, followed by the abortion law in 1997 [7]. This was followed by the introduction of the Cambodian national population policy in 2003 [7]. The FP program was made available in numerous health centres across Cambodia [7]. In 2008, a health strategic plan was integrated within the FP program, followed by the fast track initiative of 2010, which highlighted the achievements of the millennium development goals (MDGs), especially MDG 5 target 5b on reproductive health [6]. Cambodian national guidelines for adolescent and youth friendly services were introduced only a decade ago in 2008 by the Cambodian Ministry of Health for the national reproductive health program of the national maternal and child health centre [8]. The Government of Cambodia and associated partners stated their commitment to comprehensive family planning during the first “national conference on family planning” in 2014 [6]. Cambodia has been a part of the FP 2020 initiative [9]. The FP 2020 initiative was inspired by the London Summit on FP in 2012, and the goal was to have an additional 120 million adolescent girls and women to become users of modern contraceptive methods in 69 of the low- and middle-income countries (LMICs) by the year 2020 [9].

In Cambodia, the FP services provided by the government hospital staff sometimes only include patient counselling and provision of information, as there is usually a shortage of modern contraceptive methods at the hospitals (1). There is no tracking or follow up system for women who have been referred to the health centres for family planning, either in the urban or rural areas [6]. Further investigation by the World Health Organization (WHO) in rural areas in Cambodia revealed that only one in three villages had active community-based distributers for provision of oral pills and condoms [6]. In Cambodia, the majority of the FP services and modern contraceptive methods are provided by the government clinics (50%), while private clinics provide 40% of the FP services. Contraceptives acquired at different pharmacies and shops or through friends make up the remaining 10% [10].

Abortions were made legal by the revised Cambodian Penal Code in 1997 to allow termination of pregnancy on request until the 12th week of pregnancy [11]. The abortion law 1997 makes it clear that abortions can be carried out only in a hospital or health centre by medical doctors, or by midwives authorized by the Cambodian Ministry of Health [1]. However, in many Buddhist societies, including Cambodia, abortion is considered a sin [12]. Many factors act as barriers for safe abortions including practitioners’ reluctance to perform abortions, unstructured abortion fees, lack of highly trained providers, and women’s fear of ill-treatment by providers [11,12]. This results in many adolescent girls and women seeking abortion services elsewhere [12]. There is a common practice amongst unmarried young women to buy abortion medications from untrained shop keepers at local pharmacies [12]. These young women try to manage their own pregnancy termination with dire results, requiring an urgent surgical intervention that causes additional economic cost and increased risk of mortality [12].

Data from the United Nations Population Fund (UNFPA) in 2018 show that although adolescent pregnancy rates are declining at the global level; this is not the case in most countries in the South East Asian region [13]. Cambodia, the Philippines and Indonesia still report high adolescent pregnancy rates [13]. The Cambodian National Institute of Statistics (CNIS) reports that teenage pregnancies amongst adolescents aged 15–19 years have increased from 8% in 2010 to 12% in 2014, despite a drop in the total fertility rate (TFR) from 3 children to 2.7 children per woman across the same period [1].

Unintended pregnancies (UIPs) result from non-use or failure of a contraceptive method, and can be prevented by correct, consistent and effective use of contraceptive methods [14]. Analyses of the 2014 Cambodian Demographic and Health Survey (CDHS) data by the World Health Organization (WHO) showed that 10% of current pregnancies amongst 15–49 year old women were UIPs [6]. It was also found that 46% of women had not used any contraceptive methods before the UIP, 25% had used traditional methods, 26% had used short acting modern contraceptive methods, and 3% had used long-acting reversible contraceptive methods (LARCs) [6]. The WHO reports that 30% of married Cambodian women do not want to become pregnant, but they either do not use any modern contraceptive methods or use traditional methods [6], which can be less effective. This gap between the desire to not become pregnant and non-use of any contraceptive method puts these women at a higher risk of having UIPs and unsafe abortions [6].

Universal access to sexual and reproductive health, including access to contraception, is an important objective of the Sustainable Development Goals (SDGs) 2030, first introduced in 2015 by the United Nations [15]. Reproductive health is an integral part of SDG 3 for “Good health and wellbeing”, but may also be considered as an important part of SDG 5 for “Gender equality and women’s empowerment” [15]. More effort is needed for Cambodia to reduce the burden of UIPs and to meet the Target 3.7 of SDG 3 and Target 5.6 of SDG 5 [15]. Detailed information about Targets 3.7 and 5.6 with their indicators is available elsewhere [15].

### 1.1. Theoretical Framework

There is limited research available that explores the social and ecological factors that influence UIP amongst Cambodian adolescent girls (15–19 years) and women in their twenties (20–29 years). This study uses Bronfenbrenner’s social ecological model (SEM) [16] as the theoretical basis for identifying factors influencing UIP. This SEM was modified by McLeroy et al. in 1988 [17] and Koren et al. in 2010 presented a modified version of this SEM for UIPs [18]. Factors influencing UIP could be operating at the individual (intrapersonal) level, including age, knowledge, attitudes, beliefs, practices, area of residence, employment, education and wealth status; at the microenvironment (interpersonal, institutional and community) level, including partners and peers; and at the macroenvironment (policy or relevant legislation) level [16,18]. The rationale for using the SEM theoretical framework [16,18] is to provide a structural platform for logical understanding of the impact and association of multiple factors associated with UIP and to identify potential gaps in knowledge and a future research direction for policy planners and stakeholders [19].

### 1.2. Aim

The primary objective was to ascertain socioeconomic and demographic factors that influence UIP amongst sexually active females aged 15–29 years in Cambodia using Bronfenbrenner’s social ecological model (SEM).

## 2. Materials and Methods

This study used population data from the Cambodian Demographic and Health Survey (CDHS) from 2014. The 2014 CDHS is the latest and fourth nationally representative DHS. Weighted data were analysed using multiple logistic regression analyses for 3406 Cambodian sexually active single, in union or married females aged 15–29 years. An updated and comprehensive list of 28,455 eligible enumeration areas (EAs) was provided by the Cambodian National Institute of Statistics (CNIS) for 2014 CDHS [1] and each EA included approximately 119 urban residential households or 95 rural households, with an overall average of 99 households per EA [1]. Two stage-stratified sampling and probability systematic sampling methods were used to select participants [1]. The 2014 CDHS included 14 individual provinces as well as five groups of provinces to make up the 19 sampling domains and 186 primary sampling units (PSUs) [1]. It included 15,825 households, in which a total of 17,578 women (15–49 years) and 5190 men (15–49 years) were interviewed [1]. Structured questionnaires were used to collect data by experienced interviewers who had prior field training and the survey response rate was 99.8% [1]. The CNIS has published details pertaining to the sampling frame, survey design, methods, questionnaires, and participant sampling and selection process [1]. The data used in this study came from the woman’s questionnaire “DHS7-Womans-QRE-EN KHIR72FL” [1]. Females who gave a response to the question “age at first sex” were classified as sexually active.

Modern contraceptive methods include (a) short-acting reversible contraceptive methods; combined oral contraceptive pills (COCPs), progesterone only pills (POPs), male condoms and female condoms (diaphragm), and emergency hormonal contraception; (b) long-acting reversible methods include; intrauterine contraceptive devices (IUCDs), injectables, dermal implants; and (c) non-reversible permanent contraceptive methods include: female and male sterilization [1,9]. Traditional contraceptive methods include abstinence, periodic rhythm method, withdrawal method or coitus interruptus and any other folk methods reported by the respondent including tinctures, potions, and herbs [1,9].

### 2.1. Ethics

De-identified data from the 2014 Cambodian DHS is freely available from the DHS Program website [20]. We received approval to use the data from the MEASURE Demographic Health Survey (DHS) Program and an ethics exemption for data analyses from the Deakin University Human Research Ethics Committee (DUHREC), Victoria, Australia (project no. 2018-157). The 2014 CDHS reports that ethics permission was received from the Cambodian Ministry of Health, and a strict protocol of standardized processes for data collection was observed, including informed consent from all participants. Additional information is available from the Cambodian National Institute of Statistics website [8].

### 2.2. Outcome Variable (Unintended Pregnancy)

A UIP is defined and calculated as a pregnancy which is either unwanted or mistimed [21]. A woman who has an unwanted pregnancy does not want to be pregnant or have any children. A woman who has a mistimed pregnancy does not want to be pregnant at this time but wants the pregnancy later [21].

The Demographic and Health Survey (DHS) includes questions about children born in the preceding five years so that the status of UIPs can be assessed. Women were asked the following two questions about each of their pregnancies: (a) “When you got pregnant with (NAME), did you want to get pregnant at that time? (Yes/No)” [1] and (b) “Did you want to have a baby later, or did you not want any (more) children? (Yes/No)” [1]. For calculating UIP as the binary dependent variable of outcome, the statistical analyses in our study included only the current pregnancy or the most recent pregnancy within the last three to five years prior to the survey.

### 2.3. Multiple Independent Variables

Within the regression analyses the following variables were included at the individual level of the SEM [16]: age groups of female participants in years (15–19, 20–24, 25–29); area of residence (rural and urban), parity (used as a continuous variable); current use of contraceptive methods after having an unintended pregnancy (modern methods, traditional methods or no contraceptive methods); history of pregnancy termination or abortion (yes/no), woman’s education status (no education, primary, secondary, higher), current employment status (yes/no), and wealth index status [22] (poorest, poorer, middle, richer and richest) [1]. Variables under the microenvironment level included woman’s autonomy in terms of the person in the household deciding about the woman’s access to healthcare (the woman herself, woman and husband’s joint decision, husband only, someone else in the household, usually pertaining to the mother-in-law or parents-in-law). Variables under the macroenvironment level included; whether women had listened to any messages about family planning on radio or television in the previous 3–4 months (yes/no).

The “wealth index” in the CDHS was constructed using household assets data (including assets specific to country) and principle components analysis [1]. All information about calculation of wealth index is available from the household questionnaire [1] in the DHS program [20] and CNIS [8]. The socioeconomic status of households was measured by the DHS-guided calculation of wealth index [1]. The asset information concerns household ownership of several consumer items ranging from a television to a bicycle or car, as well as other characteristics such as type of drinking water available, sanitation facilities used, roofing and flooring [1]. Each asset was assigned a weight (factor score) generated through principle components analysis, and the resulting asset scores were standardized in relation to a standard normal distribution with a mean of zero and a standard deviation of one [22]. Each household was then assigned a score for each asset, and the scores were summed up by the household. The sample was weighted by the number of members in each household and then divided into population quintiles. Each quintile was designated a rank, from one (poorest) to five (richest) [1].

### 2.4. Statistical Analyses

Stata 15 SE [23] was used for descriptive, bivariate and multiple logistic regression analyses. The *p*-value was taken as statistically significant if <0.05. Pearson’s Chi square test of significance was applied to determine the degree of association between “UIP” and each categorical variable. Survey weights were applied to adjust for cluster sampling in the survey and multiple logistic regression analyses were reported as odds ratios with 95% CI for unadjusted (crude OR) and adjusted models (AOR). Crude ORs were calculated by analysing the effect of only one independent variable affecting UIP, and AORs were obtained by including all independent variables influencing UIP. Our study originally included 4823 sexually active females aged 15–29 years [24]. Upon close inspection, the hospital data registry in the dataset showed that there were 1417 missing values for the dependent variable (UIP). These 1417 women did not have any children born yet at the time of the survey. As a result, there was a skip pattern in the questionnaire, and these women chose not to answer the questions pertaining to the UIP. These 1417 missing values were listwise deleted and the total sample size for analysis was 3406 (see Table 1). To account for the missing data of 193 women in the independent variable “woman’s autonomy”, multiple imputations (MI) (20 imputations) were performed in Stata 15 SE and the final model is presented in Table 2 (*n* = 3406). Urban and rural samples were analysed separately to determine if factors associated with UIP behaved in the same way across locations.

## 3. Results

Table 1 shows the data included as 883 (26%) urban and 2523 (74%) rural females. The social demographic characteristics of the sample are presented in Table 1.

Results of the countrywide multiple logistic regression analyses (Table 2) are presented under the individual, microenvironment and macroenvironment levels of SEM [16]. After deletion of missing values, the total sample size was 3213 for which AOR were calculated (Model I in Table 2). To account for missing data for 193 women in the independent variable “woman’s autonomy”, multiple imputations (MIs) (20 imputations) were performed in Stata 15 SE. The final Model II after multiple imputations (*n* = 3406) in Table 2 shows the AOR for countrywide sample.

Urban and rural multiple logistic regression analyses including a comparison of adjusted odds ratios (AORs) are presented in Table 3.

### 3.1. Social Ecological Model—Individual Level Factors

a Knowledge and prevalence of contraceptive methods

Descriptive analyses show that 99.7% females had heard about modern and traditional contraceptive methods. Amongst females currently using contraception, 13.6% were using traditional methods and 35.3% were using modern methods.

b Correct knowledge of menstrual cycle

Females were asked whether they knew about certain days between two menstrual periods in which they could become pregnant if they were sexually active and not using any contraceptive methods. Descriptive analyses show that only 1084 participants (31.8%) answered correctly that the fertile period was halfway between two periods, and 2321 (68.2%) participants answered incorrectly or did not have any understanding of the fertility period in their menstrual cycles.

c Unintended pregnancy

A total of 3406 sexually active females aged 15–29 years were included in the sample and the overall prevalence of UIP was 12.3%. Descriptive analyses show that the prevalence of UIP was 16.3% in the urban region and 11% in the rural region. The prevalence of UIP amongst urban females was 18% in adolescents (15–19 years), 15.2% in young women aged 20–24 years, and 16.8% in women aged 25–29 years. The prevalence of UIP amongst rural females was 8.3% in adolescents (15–19 years), 9.3% in young women aged 20–24 years, and 12.8% in women aged 25–29 years.

Bivariate analyses using Chi square test show a statistically significant association between UIP and the three age groups (*p* = 0.01).

Multiple regression analyses show increased likelihood of UIP in adolescents (15–19 years) and young women (20–24 years) as compared to 25–29-year-old women (reference group) (see Model II in Table 2). There was an increased likelihood of UIP in the urban region (AOR 1.4, CI 1.01–1.8) (see Model II in Table 2). The rural model showed increased likelihood of UIP in adolescents (15–19 years) (see Table 3).

d Current use of modern contraceptive methods

In the adjusted analyses overall (Model II in Table 2), women were more likely to be currently using modern contraceptive methods if they reported that their last pregnancy (with in the last three years) was an unintended pregnancy (*p* < 0.01).

e Increased parity

There was an increased likelihood of UIP with multiparity (AOR 2.1, CI 1.8–2.4) (see Model II in Table 2). Urban and Rural models also showed increased likelihood of UIP with multiparity (total number of children born) (see Table 3).

f History of pregnancy termination

There was an increased likelihood of UIP in women with a history of abortion (AOR 1.4, CI 1.1–1.8) (see Model II in Table 2).

g Wealth Index

There was a decreased likelihood of UIP in women belonging to the poor socioeconomic status (including poorer and poorest wealth index) (Model II in Table 2).

h Current employment

Urban model showed that there was a decreased likelihood of UIP in women who were currently employed (see Table 3).

### 3.2. Social Ecological Model—Microenvironment Level Factors

a Women’s autonomy

Descriptive analyses showed that 323 (7.2%) women could not demand that their husbands use condoms at the time of sexual intercourse and 316 (7.1%) were not sure if they could do this, which potentially increases their risk for UIP.

The adjusted multiple logistic regression (Model II in Table 2) showed that there was an increased likelihood of UIP in women with decreased autonomy or decision-making ability to access health care. This happened when the decision to access health care for the woman was made by the husband alone, or someone else in the household (usually the parents-in-law or the mother in law).

### 3.3. Social Ecological Model—Macroenvironment Level Factors

a Family Planning messages on media

The countrywide model showed no association of UIP with women hearing about FP messages on media including radio and television in the past few months. However, the urban model showed significant association of UIP in women who had heard about any FP messages on the television in the last three months (*p* = 0.04).

## 4. Discussion

Our study provides important insights into the various social and demographic factors influencing unintended pregnancies in Cambodia. We found increased likelihood of UIP with younger age groups, multiparity, history of abortion, unemployment and reduced personal autonomy amongst women in terms of decreased decision making on access to healthcare.

### 4.1. Individual Level of SEM

The prevalence of UIP in 15–29 years old women was 12.3% which is slightly higher than the national level of UIP in women 15–49 years in Cambodia [6]. Our results report increased likelihood of UIP in younger women (15–24 years), which is also reflected in the rural model showing a significant association of UIP with the adolescent age group. Previous reports have identified that UIPs amongst adolescent girls and young women in Cambodia [1], as well as in many other Southeast Asian countries [25], are a result of rapid repeat pregnancies [25]. This was also reflected in our results. Marque et al. (2012) suggest that women having an UIP, as compared to those having an intended pregnancy, may be less inclined to begin pre-natal care within the first pregnancy trimester or more likely to opt for abortion [26].

Petitet (2018) [27] reports that the abortion rate in Cambodia could be one of the highest amongst countries in the South-East Asian region. We posit that in most adolescent girls or young women, fertility could return soon after they have had an abortion. These women could be at an increased risk of having an UIP if they did not use any effective contraception after an abortion. Smith et al (2015) suggest that as a woman’s fertility can return within two–three weeks post abortion, it is imperative for women to start contraception soon after an abortion to prevent any future UIPs [28]. Bearinger et al. (2007) [29] suggest a higher burden of having UIPs amongst unmarried adolescents who had induced abortions as compared to older women.

Countrywide sample shows a significant increase in women using modern contraceptive methods after experiencing an UIP. We suggest that these females may be cautious after having a UIP and looking for a more effective contraceptive method, so they start using modern methods or switch from traditional to modern methods. Petitet et al. (2010) also report current use of modern contraceptive methods in Cambodian young women only after having an UIP [30]. Kenny (2017) [3] reports similar findings in her work with adolescent mothers in Ratanak Kiri Province in Cambodia. Sreytouch (2008) [7], in a study in Banteay Meanchay, Cambodia, reports that 18% of women of child bearing age start using modern contraceptive methods after having their first baby because they want to avoid any future UIPs. These women from Banteay Meanchay started using modern methods as a form of a better contraceptive cover so they could prevent the low standard of living exacerbated by the increased financial cost of an additional child [7]. McDougall et al. (2009) [31] recommend increasing the post-abortion access to modern contraceptive methods at the health centres for Cambodian women seeking pregnancy termination or abortion services which may reduce their chances of subsequent UIPs and unsafe abortions in future.

There was a decreased likelihood of UIP in currently employed urban females. This may suggest that meaningful, waged employment along with improved sexual and reproductive health (SRH) education in young women can reduce the gap in knowledge and improve behaviour pertaining to the consistent and effective contraceptive use. Haffejee et al. (2017) in South Africa reported that current employment status improved women’s autonomy to use contraceptive methods [32]. Our study suggests that women working in full-time employment may have wanted to space or limit their future pregnancies, especially in urban families. Sawyer et al. (2012) [33] suggest that the reasons for UIP could include low literacy levels due to the fact of interrupted education and unemployment. Women having a UIP can enter into a vicious cycle of poor economic opportunities, which result in reduced lifetime earnings, leading to decreased personal, reproductive and economic autonomy in women [33].

### 4.2. Microenvironment Level of SEM

Females with lower autonomy are more likely to experience UIP when the only the husband or someone else in the household makes decision for their access to healthcare. Studies from countries in Asia and Africa show that the decision to access reproductive health care in women is usually made either by their husbands or by another family member in the household who is most likely their mother-in-law or parents-in-law [34]. Sreytouch (2008) [7] from Cambodia and Chandra-Mouli et al. (2014) [35] from other LMICs suggest that women who marry early may not use contraception as they face pressure from their husbands or mothers-in-law to bear children [7,35]. Hence, a woman’s preference for contraception may not be considered, resulting in UIPs [7]. Current literature provides ample evidence of the association between women’s optimum reproductive health and their autonomy to access healthcare and gain empowerment and gender equity [36,37].

Although the association of UIPs and poor wealth status has mixed results from the literature review [36], our results show decreased likelihood of UIP in women belonging to the poor socioeconomic status. We posit that women from low socioeconomic status were cognizant of the financial difficulties of raising a child, hence they were more likely to use effective contraception to reduce the risk of having UIPs. As we have reported earlier, there is an increased likelihood of UIP with a history of abortion. Perhaps these women from a low socioeconomic status already had an abortion, and they became more concerned about preventing any subsequent UIPs. A similar explanation is reported in another study from Nairobi, Kenya [38].

### 4.3. Macroenvironment Level of SEM

There was an increased likelihood amongst urban women to recognise that they probably had an UIP in the past three years if they had heard any FP messages on the television recently (three months ago). As these women became aware of the FP messages, they probably recognized that their last pregnancy in the past three years was an UIP. This could be an opportunity for potential implementation of a targeted and holistic SRH program through the media to increase women’s and couples’ awareness about UIPs and use of effective contraception.

In Cambodia, FP messages are broadcast by the government through media and are usually accessed via television (51%) and radio (38%) with lesser access through the print media (17%) [1]. The United Nations Population Fund (UNFPA) has been facilitating a local organization, the Reproductive Health Association of Cambodia (RHAC), to run a mobile-based FP text messages campaign. This campaign includes SRH information and is designed to reach rural women in four operational districts of Takeo province [39]. As reported by Matsouka et al. (2010) [40] in a study in rural Cambodia, there is limited knowledge about FP and reproductive health services amongst rural women. A survey was conducted [7] to assess the knowledge of women in Banteay Meanchay, the 13th largest province in Cambodia, and 99.3% had knowledge of modern contraceptive methods regardless of their educational and socioeconomic status. The study also reported that information about FP was mostly obtained via healthcare staff (67.4%), followed by television (50.7%), radio and friends (33%), non-governmental organizations’ pamphlets (7%), relatives (3.6%), and teachers at school (1.4%) [7].

Cambodia did not have any formal adolescent reproductive health (ARH) policy or a multi-sectoral policy on youth until 2008 [8]. There has been no formal SRH literacy program for youth as a part of the education curriculum until the Country Program Action Plan 2016–2018, which was developed by UNFPA and the Royal Government of Cambodia [41]. Only future data will show if this initiative is successful at reducing UIPs and abortions in adolescent girls and young women.

## 5. Conclusions

Limited access to modern contraceptive methods, low literacy about SRH issues, and decreased autonomy is universal in females living in Cambodia. Multiple factors influence UIP which include younger age groups, multiparity, history of abortion, unemployment and decreased personal autonomy. A high rate of UIP is an indicator for low contraceptive use and, subsequently, a measure of unmet need for contraception. These data may inform future sexual and reproductive health policy.

## 6. Recommendations

There is a need for designing and implementing national level policies to raise awareness about safe sex and correct, consistent use of modern contraceptive methods including oral hormonal pills and condoms as well as long-acting reversible contraceptives (LARC). There is a need to promote LARCs which include subdermal implants and intrauterine contraceptive devices (IUCDs). Multi-pronged interventions are required to increase accessibility to modern contraceptive methods and to raise awareness about women’s sexual and reproductive health rights. A holistic and culturally appropriate intervention is needed to raise Cambodian women’s autonomy at the individual, household and society levels by targeting youth and married couples and the elders in the society. More quantitative and qualitative research is required to inform potential intervention strategies to reduce unintended pregnancies. A comprehensive campaign could improve reproductive health indicators and lead to educational and economic gains for the household and community.

## 7. Limitations

The study has used nationally representative data from the latest 2014 CDHS. Unintended pregnancy is typically underreported in Cambodia, and few international comparisons exist as prospective data on UIPs is not collected in most countries. As our study is cross-sectional, causality cannot be established but the results describe associations between various factors and UIP. The study’s focus was on Cambodian female youth, as the negative personal and social consequences of UIP and unsafe abortions are borne by the females. However, additional information may be gathered on risks for UIP from males in the same age groups in future studies. The CDHS data is self-reported and may be subject to recall bias when collecting information about women’s last pregnancy. In addition, women may become unsure about their feelings of mistimed or unwanted pregnancy once the baby has been born and may rationalize any previous negative feelings associated with the UIP. For our analyses for UIPs, only the current pregnancy or the last pregnancy was included which resulted in a live birth within three to five years prior to 2014 CDHS survey.

## Figures and Tables

**Table 1:** Percent distribution of sociodemographic details of Cambodian urban and rural sexually active, single, in union or married females 15–29 years old, the 2014 Cambodian Demographic and Health Survey (CDHS) (urban *n* = 883, rural *n* = 2523, total = 3406).

No	Sociodemographic Details	Age Group 15–19 Years (*n* and %)		Age Group 20–24 Years		Age Group 25–29 Years	
				(*n* and %)		(*n* and %)	
		Urban	Rural	Urban	Rural	Urban	Rural
	Individual Level of Social Economic Model ^a^						
1	Number of participants	50	181	309	1074	524	1268
		5.7%	7.2%	35%	42.6%	59.3%	50.2%
2	Education level						
	No education	2	21	19	99	20	193
		4%	11.6%	6.5%	9.2%	3.8%	15.2%
	Primary	21	89	98	521	160	673
		42%	49.8%	31.7%	48.5%	30.5%	53%
	Secondary	27	71	169	447	255	380
		54%	39.2%	54.7%	41.6%	48.6%	30%
	Higher	0	0	23	7	89	22
		0%	0%	7.4%	0.6%	16.9%	1.7%
3	Marital status						
	Married	45	167	283	1015	490	1199
		90%	92.2%	91.6%	94.5%	93.5%	94.5%
	living with a partner	0	0	1	7	4	6
		0%	0%	0.3%	0.6%	0.7%	0.5%
	Widowed/Divorced/no longer living together	5	14	25	52	30	63
		10%	7.8%	8%	4.9%	5.7%	5%
4	Current employment status						
	Yes	19	94	177	663	383	851
		38%	52%	57.3%	61.7%	73%	67.1%
	No	31	87	132	411	141	416
		62%	48%	42.7%	38.2%	27%	32.8%
5	Wealth index						
	Poorest	22	48	107	270	139	295
		45%	26.8%	35.2%	25.4%	27%	23.4%
	Poorer	10	51	78	216	112	269
		20.4%	28.5%	25.6%	20.3%	21.7%	21.4%
	Middle	10	31	52	215	100	241
		20.4%	17.3%	17.1%	20.2%	19.4%	19.2%
	Richer	4	22	47	180	89	243
		8.1%	12.3%	15.4%	17%	17.2%	19.3%
	Richest	3	27	20	180	76	209
		6.1%	15.1%	6.5%	17%	14.7%	16.6%
6	Number of children ever born/parity						
	1	47	164	227	746	221	410
		94%	90.6%	73.4%	69.4%	42.2%	32.3%
	2	3	16	74	282	228	547
		6%	8.8%	24%	26.6%	43.5%	43.1%
	3	0	1	7	41	67	222
		0%	0.5%	2.2%	3.8%	12.8%	17.5%
	4	0	0	1	5	6	73
		0%	0%	0.2%	0.5%	1.1%	5.7%
	>4	0	0	0	0	2	16
		0%	%	0%	%	0.4%	1.3%
7	Status of current contraceptive use after an unintended pregnancy						
	No contraceptive use	31	103	142	457	183	483
		62%	57%	46%	42.5%	35%	38.1%
	Traditional contraceptive methods	8	8	62	137	135	184
		16%	4.4%	20%	12.7%	25.7%	14.5%
	Modern contraceptive methods	11	70	105	480	206	601
		22%	38.7%	34%	44.7%	39.3%	47.4%
	Microenvironment Level of Social Ecological Model ^a^						
8	Women’s autonomy—the person who decides for the respondent’s access to healthcare						
	Woman herself	12	74	116	433	193	524
		26.6%	44.6%	41%	42.4%	39.1%	43.5%
	Joint decision of husband and respondent	28	81	139	496	249	612
		62.2%	48.8%	49.1%	48.5%	50.4%	50.8%
	Husband only	4	7	26	85	47	65
		8.9%	4.2%	9.2%	8.3%	9.5%	5.4%
	Someone else	1	4	2	8	5	4
		2.2%	2.4%	0.7%	0.7%	1%	0.3%
	Macroenvironment Level of Social Ecological Model ^a^						
9	Participants heard about family planning messages on radio in the last few (3–4) months						
	Yes	12	59	124	376	208	465
		24%	32.6%	40.1%	35%	39.7%	36.7%
	No	38	122	185	697	316	803
		76%	67.4%	59.8%	65%	60.3%	63.3%
10	Participants heard about family planning messages on television in the last few (3–4) months						
	Yes	60	64	329	432	519	545
		55.5%	35.3%	67.3%	40.2%	71%	43%
	No	48	117	160	641	213	723
		44.4%	64.6%	32.7%	59.8%	29%	57%

^a^ Three levels of Bronfenbrenner’s social ecological model (SEM) as the theoretical basis for identifying factors influencing unintended pregnancy in Cambodian females 15–29 years old.

**Table 2:** Multiple logistic regression analyses model for factors influencing unintended pregnancy in sexually active, single, in union or married Cambodian females 15–29 years old (countrywide model).

Factors Influencing Unintended Pregnancy at Countrywide Level	Proportions Unintended Pregnancy (Yes)	Crude Odds Ratio (OR) and 95% Confidence Interval (CI) with *p*-Values	Adjusted Odds Ratio (AOR) and 95% Confidence Interval (CI) with *p*-Values, *n* = 3213, Model II before Multiple Imputations	Adjusted Odds Ratio (AOR) and 95% Confidence Interval (CI) with *p*-Values, *n* = 3406, Model II after Multiple Imputations
Individual Level of Social Ecological Model				
Region				
Urban	16.3%	2 (1.5–2.5), *p = 0.001	1.6 (1.1–2.3), *p = 0.01	1.4 (1–1.8), * *p* = 0.04
Rural (base)	11%			
Age Group				
15–19 years	10.4%	0.9 (0.5–1.5), *p* = 0.7	2.2 (1.1–4.1), *p = 0.01	1.6 (1–2.7), * *p* = 0.04
20–24 years	10.6%	0.7 (0.5–0.9), *p = 0.01	1.2 (0.8–1.6), *p* = 0.3	1.3 (1–1.6), * *p* = 0.04
25–29 years (base)	14%			
Education				
No education	11.3%	1.2 (0.5–2.7), *p* = 0.6	0.9 (0.4–2.3), *p* = 0.9	0.8 (0.4–1.7), *p* = 0.6
Primary	13%	1.5 (0.7–3.2), *p* = 0.2	1.6 (0.7–3.3), *p* = 0.2	1.2 (0.7–2.2), *p* = 0.4
Secondary	12%	1.2 (0.6–2.6), *p* = 0.5	1.7 (0.7–3.7), *p* = 0.2	1.2 (0.7–2.2), *p* = 0.5
Higher (base)	10.6%			
Parity		1.9 (1.6–2.2),*p = 0.001	2.2 (1.8–2.6),*p = 0.001	2.1 (1.8–2.4),*p = 0.001
Current contraceptive use after having an unintended pregnancy				
Traditional methods	12%	1.3 (0.9–1.9), *p* = 0.2	1.2 (0.7–1.7), *p* = 0.4	1 (0.7–1.4), *p* = 0.9
Modern methods	14.2%	1.3 (0.9–1.7), *p* = 0.08	1.3 (0.9–1.7), *p* = 0.1	1.4 (1–1.7), * *p* = 0.009
No contraceptive use (base)	10.5%			
History of pregnancy termination				
Yes	17.3%	1.6 (1.2–2.2),*p = 0.002	1.4 (1–1.9), *p = 0.05	1.4 (1.1–1.8), *p = 0.002
No (base)	10.8%			
Current employment				
Yes	11.8%	0.8 (0.6–1.01),*p* = 0.09	0.7 (0.5–1.03), *p* = 0.07	0.8 (0.7-1), p = 0.2
No (base)	13.3%			
Wealth Index				
Poorest	10%	0.6 (0.4–0.9), *p = 0.01	0.6 (0.3–1.04), p=0.06	0.5 (0.3–0.7), *p = 0.001
Poorer	11.4%	0.6 (0.4–0.9), *p = 0.02	0.6 (0.4–1.09), *p* = 0.1	0.6 (0.4–0.9), *p = 0.02
Middle	11.6%	0.6 (0.4–0.9), *p = 0.01	0.7 (0.4–1.05), *p* = 0.08	0.7 (0.5–1), *p* = 0.09
Richer	11.8%	0.6 (0.4–0.9), *p = 0.02	0.7 (0.4–1.1), *p* = 0.1	0.7 (0.5–1), *p* = 0.07
Richest (base)	16.2%			
Microenvironment Level of Social Ecological Model				
Person deciding woman’s access to healthcare				
Respondent and husband/partner	11.7%	1 (0.8–1.4), *p* = 0.7	1.1 (0.8–1.4), *p* = 0.7	1 (0.8–1.3), *p* = 0.9
Husband/partner alone	18.4%	1.5 (0.9–2.2), *p* = 0.06	1.3 (0.8–2.1), *p* = 0.2	1.7 (1.1–2.5), *p = 0.008
Someone else in the family	29.2%	2.8 (1–7.8), *p = 0.04	3.2 (1.1–8.8), *p = 0.02	3.7 (1.5–9.5), *p = 0.005
Respondent alone (base)	11.5%			
Macroenvironment Level of Social Ecological Model				
Participants heard about family planning messages on radio in the last few (3–4) months				
Yes	11.2%	0.7 (0.5–0.9), *p = 0.02	0.8 (0.6–1.2), *p* = 0.3	0.9 (0.7–1.2), *p* = 0.7
No (base)	13%			
Participants heard about family planning messages on television in the last few (3–4) months				
Yes	12%	0.8 (0.6–1.1), *p* = 0.2	0.8 (0.6–1.2), *p* = 0.3	0.8 (0.6–1), *p* = 0.2
No (base)	12.7%			

Number of observations in the final Model II = 3406; *p*-value * significant if <0.05.

**Table 3:** Multiple logistic regression analyses showing comparison of adjusted odds ratios (AOR) in urban and rural models (without multiple imputations) for factors influencing unintended pregnancy amongst Cambodian sexually active, single, in union or married females (15–29 years).

Factors Influencing Unintended Pregnancy	Adjusted Odds Ratio (AOR) (95% CI) with *p*-Values Urban Model (*n* = 811) *^#^	Adjusted Odds Ratio (AOR) (95% CI) with *p*-Values Rural Model (*n* = 2366) *^##^
Individual Level of Social Ecological Model		
Age Group		
15–19 years	2.6 (0.9–7.3), *p* = 0.07	2 (1–4.3), * *p* = 0.04
20–24 years	1.3 (0.7–2.7), *p* = 0.4	1.1 (0.7–1.6), *p* = 0.6
25–29 years (base)		
Education		
No education	0.9 (0.1–6.9), *p* = 0.9	1.7 (0.2–15), *p* = 0.6
Primary	1.3 (0.4–4.1), *p* = 0.6	3.1 (0.4–25.7), *p* = 0.3
Secondary	1.4 (0.6–3.1), *p* = 0.4	2.8 (0.3–23.1), *p* = 0.3
Higher (base)		
Parity (continuous variable)	2.1 (1.3–3.4), * *p* = 0.002	2.2 (1.8–2.6), * *p* = 0.001
Current use of contraceptive methods after having an unintended pregnancy		
Traditional methods	1 (0.5–2.2), *p* = 0.9	1.2 (0.7–1.9), *p* = 0.4
Modern methods	1.4 (0.8–2.5), *p* = 0.2	1.2 (0.8–1.8), *p* = 0.2
No contraceptive methods (base)		
History of pregnancy termination		
Yes	1.8 (0.2–3.8), *p* = 0.1	1.3 (0.8–1.9), *p* = 0.2
No (base)		
Employment		
Yes	0.6 (0.3–1), * *p* = 0.05	0.8 (0.5–1.2), *p* = 0.4
No (base)		
Wealth Index		
Poorest	0.7 (0.3–1.5), *p* = 0.3	0.7 (0.4–1.2), *p* = 0.2
Poorer	1.4 (0.6–3.2), *p* = 0.3	0.6 (0.4–1.2), *p* = 0.1
Middle	1.1 (0.4–2.9), *p* = 0.7	0.6 (0.4–1), *p* = 0.08
Richer	2 (0.8–4.8), *p* = 0.09	0.8 (0.5–1.4), *p* = 0.4
Richest (base)		
Microenvironment Level of Social Ecological Model		
Person deciding woman’s access to healthcare		
Respondent and husband/partner	1.3 (0.8–2.4), *p* = 0.2	0.9 (0.7–1.4), *p* = 0.9
Husband/partner alone	1.8 (0.8–4.1), *p* = 0.1	1.3 (0.7–2.3), *p* = 0.4
Someone else in the family	3.8 (0.9–16.4), *p* = 0.07	3.4 (0.1–13.1), *p* = 0.06
Respondent alone (base)		
Macroenvironment Level of Social Ecological Model		
Participants heard about family planning messages on radio in the last few (3–4) months		
Yes	0.8 (0.5–1.5), *p* = 0.6	0.8 (0.5–1.3), *p* = 0.5
No (base)		
Participants heard about family planning messages on television in the last few (3–4) months		
Yes	1.9 (1–3.6), * *p* = 0.04	0.7 (0.5–1), *p* = 0.1
No (base)		

*^#^ Urban model: Number of strata = 19; number of PSUs = 183; number of observations = 811, degrees of freedom (DFs) = 164, prob > F = 0.0000; *^##^ Rural model: Number of strata = 19; number of PSUs = 419; number of observations = 2366, DFs = 400, prob > F = 0.000; *p*-value * significant if <0.05.
